# Job satisfaction of certified primary care physicians in rural Shandong Province, China: a cross-sectional study

**DOI:** 10.1186/s12913-019-3893-8

**Published:** 2019-01-28

**Authors:** Jingliang Gu, Tianmin Zhen, Yan Song, Lingzhong Xu

**Affiliations:** 1 Department of Social Medicine and Health Management, School of Public Health, Shandong University, 44 Wenhuaxi Road, Jinan, Shandong Province 250012 People’s Republic of China; 2 Shandong Institute of Medicine and Health Information, Shandong Academy of Medical Sciences, 18877 Jingshi Road, Jinan, Shandong Province 250062 People’s Republic of China

**Keywords:** Job satisfaction, Primary health care facility, Certified physician, Cross-sectional study, Shandong P rovince

## Abstract

**Background:**

The purpose of this study was to measure the level of job satisfaction of certified physicians in rural primary health care facilities (PHCFs) in Shandong Province in order to ascertain the key factors affecting their satisfaction and to provide effective information for policy decisions.

**Methods:**

This cross-sectional study was conducted among certified physicians in PHCFs in rural Shandong from June to August 2016. An anonymous questionnaire was completed by 495 participants (valid response rate: 91.6%). Data were analyzed using an exploratory factor analysis (EFA), one-way analysis of variance (ANOVA), and multiple linear regression.

**Results:**

The participants consisted of 310 (62.6%) males and 185 (37.4%) females. The overall mean score for job satisfaction among respondents was 3.41 (standard deviation (SD) 0.68), which indicated that certified physicians were partially satisfied with their jobs. Results also indicated that factors for the highest level of satisfaction among certified physicians were the internal environment and job description. Moreover, physicians were more satisfied with competency behaviours and organizational management than with working conditions and job rewards. In contrast, physicians were dissatisfied with the external environment to an extent. Overall job satisfaction decreased with more years of service. Older physicians were less satisfied with their jobs than younger ones. Physicians with a higher level of education or senior professional title were less satisfied with their jobs than those with a lower level of education or junior professional tilte. Organizational management and the external environment were the most important factors influencing job satisfaction.

**Conclusion:**

Certified physicians working in PHCFs in rural Shandong had a slightly higher level of overall job satisfaciton than usual. After recent healthcare reforms, the job satisfaction of primary health care physicians in Shandong has changed little in comparison to that of physicians in other provinces in China. More attention should be paid to the impacts of these variables (age, educational background, technical title, monthly salary, form of employment, and years of service) on job satisfaction. Numerous recommendations may be considered to enhance organizational management and the external environment. The Government should enhace the formulation, implementation, and evaluation of policies to ensure that physicians continue to enjoy working in PHCFs. In short, the Government should pay more attention to protecting the legitimate rights and interests of primary care physicians when devising medical reforms.

**Electronic supplementary material:**

The online version of this article (10.1186/s12913-019-3893-8) contains supplementary material, which is available to authorized users.

## Background

In early 2009, the Government of China (GOC) launched a new health care system reforms. The goal of this program is to establish a basic medical and health system covering both urban and rural residents, to provide safe, effective, convenient, and inexpensive medical health services for all [1]. For the last decade, China has been working to achieve universal health coverage as defined by the World Health Organization [2–4]. China’s medical and health care system has a dual structure in urban and rural areas, and there are large gaps in medical care in both areas. By the end of 2017, there were 576 million people living in rural areas while there were 4.28 medical and technical personnel per 1000 population in those areas, compared to 10.87 in urban areas. The key aspects of and difficulties with Chinese health care reforms lie in rural areas and PHCFs.

The service capacity of PHCFs needs to be enhanced and the system of primary medical and health care needs to be improved. In recent years, the GOC has emphasized that medical reform should begin at the local level and it has implemented a series of policies, such as enhancing the establishment of local basic medical and health service networks and urban community health service centres, implementing a national essential medicine system, promoting the reform of county hospitals, accelerating the training of general practitioners, advancing hierarchical diagnosis and treatment, and so on. Furthermore, the GOC provided 12 kinds of basic public health services to all residents free of charge. The national subsidy per person for basic public health services increased from 15 Chinese Yuan (CNY) in 2009 to 55 CNY in 2017. The financial subsidy per person for resident medical insurance increased from 120 CNY in 2010 to 450 CNY in 2017. In addition, the proportion of personal health expenditures to total health expenditures has dropped to less than 30% in 2017.

Despite numerous efforts, however, primary health care has not been significantly improved as expected. PHCFs have not served as a gate-keeper in terms of disease prevention and control [1, 5]. Many rural residents preferred to see a doctor in an urban hospital or tertiary hospital directly rather than in a local PHCF. Therefore, the number of outpatients and inpatients in PHCFs have not increased significantly. The “drug-dependent” medical system has not fundamentally changed, and the unbalanced distribution of medical resources is still a major concern [6]. Although expansion of health insurance coverage has led to increase in insurance reimbursements, it has not reduced the financial burden of illness borne by households, nor has it improved the quality of care [1, 7, 8]. According to statistics, personal health expenditures increased from 657.1 billion CNY in 2009 to 1513.3 billion CNY in 2017. The two popular Chinese phrases “kan bing nan” and “kan bing gui” could reflect the current reality.

Talent is the key component to developing PHCFs, and certified physicians are the most valuable resources. To solve medical difficulties for local residents, they need to receive appropriate medical care from physicians. To gain insight into possible problems, the job satisfaction of certified primary care physicians needs to be studied. Job satisfaction is not only closely related to physicians’ feelings towards a job and to the quality and efficiency of their services but is also an important indicator in evaluating retention, motivation, and work performance. Job dissatisfaction could lead to other negative consequences, such as poor communication between doctors and patients, poor quality of care, and even medical disputes [9]. In turn, it may affect the successful implementation of new health system reforms [10–12]. A previous study found a correlation between doctors’ job satisfaction and patients’ satisfaction with the quality of health care [13]. Good job satisfaction will enable patients to spend less and achieve better results [14]. Physicians with higher job satisfaction were likely to provide more satisfactory care and more effective treatment than those with lower job satisfaction [14, 15]. The existing challenge is how to create conditions to improve job satisfaction so that physicians are willing to stay in local PHCFs.

The aim of the current study was to evaluate the job satisfaction of physicians in rural Shandong Province following recent health system reforms and to identify the factors affecting their job satisfaction. This study also considered what the Government should do to help PHCFs retain talented personnel.

### Review of literature on job satisfaction of physicians

Job satisfaction is a measure of people’s contentedness with their job and an assessment of their work experience [16]. It can be measured using cognitive, affective, and behavioural components. Many researchers have provided definitions of job satisfaction. One of the most widely used is job satisfaciton defined as “a pleasurable or positive emotional state resulting from one’s work or work experiences” [17], while Motowidlo (1996) suggested that it is a perception of the “favourableness of the working environment” [18]. Attempting to combine both approaches, Brief (1998) defined job satisfaction as “an internal state that was expressed by affectively or cognitively evaluating an experienced job with some degree of favour or disfavour” [19]. Other researchers simply defined it as an individual’s satisfaction with one’s job, indicating whether or not he or she likes the job [16]. Numerous studies have revealed the importance of employee satisfaction. A study by Schneider and Bowen indicated that customer satisfaction was largely influenced by the attitudes and behaviours of service employees, while employee satisfaction was positively related to service quality [20]. Consequently, motivating and retaining qualified employees are crucial to the further success of service organizations [21]. To enhance customers’ perceptions of the quality of service, several studies have suggested that managers must increase employees’ self-efficacy and job satisfaction and reduce their role conflict and ambiguity [22, 23].

Several studies have examined the job satisfaction of physicians. A survey conducted by the American Physical Therapy Association in 1989 indicated that physicians were satisfied with their work [24]. The highest job satisfaction was the result of “autonomy,” whereas the lowest was the result of “reward”. This finding was consistent with the results of one study of doctors in Utah and another of doctors in Texas [25, 26]. Moreover, these two studies also revealed that the key factors contributing to physicians’ willingness to leave were heavy clinical workload, high patient expectations, and the clerical aspects of work. A study in New Zealand indicated that general practitioners had a rather high level of job satisfaction [27]. However, health care reforms, long working hours, too much bureaucracy and too many restrictions made them feel stressed and dissatisfied. Similar results were reported in a study of Pakistani public health professionals [28]. Working conditions, job description, and time pressures were the major reasons for dissatisfaction. Other factors influencing the level of job satisfaction were a low salary, lack of training opportunities, inadequate supervision, and insufficient financial incentives [28, 29].

According to an Australian study, lower job satisfaction was associated with poor health, training outside of Australia, fewer professional development opportunities, and longer working hours [10]. Meanwhile, lower job satisfaciton was also associated with a desire to reduce one’s working hours and an intention to leave medical profession. In contrast, a study in Lithuania found that doctors working in PHCFs had a relatively low level of overall job satisfaction [30]. The key factors influencing their dissatisfaction were compensation, social status, and workload.

The job satisfaction of physicians has garnered increasing attention among Chinese researchers. Numerous studies have discussed the factors infulencing job satisfaction and turnover intention. In Xinjiang and Anhui Provinces, Liu et al. (2010) conducted a quantitative study among township health centre (THC) employees in poor rural China. That study indicated that employees in THCs had a moderate level of job satisfaction. They were more satisfied with job significance, job competency, and teamwork than job rewards and working conditions [14]. In Hubei Province, more than a third (36.8%) of village doctors considered leaving their current positions voluntarily [31]. According to that study, income satisfaction, organizational policy implementation, wages and workload, career development opportunities, and working conditions were significant factors that contributed to the intention to quit among village doctors. This fingding was consistent with the results of a study of general practitioners in Hebei Province [32]. In Guangdong Province, the intention to quit among physicians was directly and negatively related to job satisfaction, and it was positively related to work stress and work–family conflict [33]. Thus, reducing working hours, increasing salaries, providing more opportunities for career development and training, and encouragement of senior managers may contribute to a reduction in their intention to quit. In Chongqing, a study found that 42.3% of primary care doctors intended to quit [34]. Findings suggested that the Government should increase its financial input to PHCFs, especially those in less-developed areas, and reform incentives to improve the job satisfaction among primary care physicians. A study in Liaoning indicated that Chinese community health workers enjoyed a moderate level of job satisfaction [35]. That study corroborated the evidence that stress and burnout are important predictors of intrinsic and extrinsic job satisfaction. Studies have examined the job satisfaction among rural village doctors, THC employees, community health workers, and physicians in urban state-owned medical facilities in other provinces of China [14, 36]. Their conclusions indicated that the respondents also enjoyed a moderate level of job satisfaction and that they were increasingly quitting or intending to quit because of job dissatisfaction.

Few investigations have been conducted to ascertain the job satisfaction of certified physicians in PHCFs in Shandong province following China’s recent health system reforms. Accordingly, the current study has been conducted in order to help policymakers to improve strategies designed to enhance physicians’ job satisfaction.

## Methods

### Study design

This study was designed to analyse current working conditions and to measure the job satisfaction of certified physicians working in PHCFs in rural Shandong in order to ascertain the factors influencing job satisfaction.

### Sampling

Shandong Province, one of China’s typical densely populated and economically developed regions, governs 17 prefectural-level cities. It has a good medical personnel foundation. By the end of 2016, there were 643,000 health care technicians in Shandong Province, of whom 245,000 were certified physicians, including 126,000 physicians working in rural areas. There is uneven economic development and an imbalance in medical resources among the eastern, middle, and western parts of Shandong Province. Therefore, a thorough survey of the job satisfaction of certified physicians in Shandong would provide general and representative data. Stratified random sampling was used in this study. It is a fact that the economy of Shandong is more developed in the east and less towards the west. First, two prefectural-level cities were randomly selected from the economically developed areas in the east, the developing areas in the middle, and the less-developed areas in the west, for a total of 6 prefectural-level cities. Second, two counties in each city were randomly selected, for a total of 12 counties. Finally, 12 CHs and 30 THs were randomly selected in those counties. In total, 540 copies of a questionnaire were distributed to certified physicians at the selected PHCFs. Trained research assistants distributed and collected the questionnaire. The survey was conducted from June to August 2016. Valid responses were received from 495 physicians (valid response rate: 91.6%). The questionnaire was anonymous and all participants provided informed consent prior to the study.

### Instrument

A review of the literature indicated that many instruments have been used to investigate the job satisfaction of different groups of people, such as village doctors, THC employees, and community health workers [31, 36–41]. However, those tools were not conductive to the aim of the current study. The features of questonnaires were adapted and revised, and a new one (see Additional file 1 for the questionnaire) was developed [42–44]. This questionnaire, consisting of 48 items in two parts, was used to evaluate the job satisfaction of certified primary care physicians. The first part includes socio-demographic information, such as gender, age, marital status, educational background, technical title, monthly salary, form of employment, years of service, hospital category and hospital grade. The second part consists of 38 items to assess job satisfaction, covering organizational management, the internal environment, the external environment, working conditions, competency behaviours, job rewards, and job description. These aspects are measured on a 5-point scale ranging from 1 to 5, where “1” represents extremely dissatisfied, “2” represents dissatisfied, “3” represents neither satisfied nor dissatisfied, “4” represents satisfied, and “5” represents extremely satisfied. Reliability statistics indicated a weak correlation among these items, and a regression analysis revealed no multicollinearity. The reliability coefficient (Cronbach’s alpha) was 0.945.

### Data analysis

The software packages EpiData 3.1 and IBM SPSS Statistics Version 21 were used for data entry and statistical analysis. EFA was used to identify common factors that cover all of the aspects of job satisfaction according to principal component analysis (PCA) and varimax rotation. The basic socio-demographic characteristics were summarized using descriptive statistical analysis. Different groups of physicians were compared via one-way ANOVA and a post-hoc test of least-significant differences (LSD). Pearson correlation coefficients were calculated to determine the relationship between general job satisfaction and each individual dimension. The aggregated factors influencing job satisfaction were analyzed using multivariate linear regression. A *P* value < 0.05 was considered statistically significant.

## Results

### Socio-demographic characteristics

In total, 495 certified physicians in rural Shandong Province participated in this study, including 310 (62.6%) males and 185 (37.4%) females. Among the respondents, 291 physicians (58.8%) worked in township hospitals (THs), and 204 (41.2%) worked in county hospitals (CHs). The average age of the participants was 37.78 years. Most respondents (61.8%) were university graduates, 31.7% were junior college or technical school graduates, and a small proportion (6.5%) were recipients of a master’s degree. Of the respondents, 86.7% were permanent full-time employees. In addition, 87.3% of participants had a junior technical title while only 12.7% of physicians had a senior technical title. Most (88.6%) of the physicians surveyed had a monthly salary between 2000 and 5000 CNY. However, 4% earned less than 2000 CNY a month and 8% earned more than 5000 CNY. Further details on the participants are shown in Table 1.

**Table 1:** Socio-demographic characteristics of doctors (*n* = 495)

Characteristics	Category	No. (%)
Gender	Male	310 (62.6)
	Female	185 (37.4)
Age	Younger than 25	16 (3.2)
	26–35	159 (32.1)
	36–45	260 (52.5)
	Over 46	60 (12.2)
Marital status	Not married	40 (8.1)
	Married	455 (91.9)
Educational background	Technical school graduate	25 (5.0)
	Junior college graduate	132 (26.7)
	University graduate	306 (61.8)
	Recipient of a master’s degree	32 (6.5)
Technical title	Medical Assistant	47 (9.5)
	Resident Physician	132 (26.7)
	Attending Physician	253 (51.1)
	Associate Chief Physician	57 (11.5)
	Chief Physician	6 (1.2)
Monthly salary (CNY)	Less than 2000	36 (7.4)
	2001–3000	163 (32.9)
	3001–4000	207 (41.8)
	4001–5000	69 (13.9)
	Over 5001	20 (4.0)
Form of employment	Temporary/casual	66 (13.3)
	Permanent full-time	429 (86.7)
Years of service	Fewer than 5	103 (20.8)
	6–10	88 (17.8)
	11–15	77 (15.6)
	16–20	138 (27.9)
	21–25	59 (11.9)
	More than 26	30 (6.0)
Hospital category	TH	291 (58.8)
	CH	204 (41.2)
Hospital grade	Grade I hospital	282 (57.0)
	Grade II hospital	213 (43.0)

### Factor analysis

An EFA was conducted using a correlation matrix with varimax rotation. There were 7 factors with eigenvalues greater than 1.0. Eigenvalues measure how much the factors explian the observed components. The initial eigenvalues indicated that the factors explained from 2.841 to 38.379% of the variance. As a result, these 7 factors were selected, with a cumulative initial eigenvalue of 65.804% and a Kaiser-Meyer-Olkin (KMO) value of 0.944 (Table 2).

**Table 2:** Total variance explained by each factor

Factor	Initial eigenvalues			Extraction sums of squared loadings			Rotation sums of squared loadings		
	Total	% of variance	Cumulative %	Total	% of variance	Cumulative %	Total	% of variance	Cumulative %
1	14.584	38.379	38.379	14.584	38.379	38.379	7.226	19.017	19.017
2	3.295	8.671	47.049	3.295	8.671	47.049	4.285	11.276	30.293
3	1.871	4.925	51.974	1.871	4.925	51.974	4.111	10.819	41.112
4	1.552	4.083	56.058	1.552	4.083	56.058	2.573	6.772	47.884
5	1.394	3.667	59.725	1.394	3.667	59.725	2.438	6.416	54.301
6	1.230	3.238	62.962	1.230	3.238	62.962	2.294	6.036	60.337
7	1.080	2.841	65.804	1.080	2.841	65.804	2.077	5.467	65.804

### Common factors for job satisfaction

Table 3 shows the categories of survey questions and their associated factor loadings. Six questions were not closely associated (greater than 0.5) with any factors (Q32: Competence improved, Q46: Recognition by the patient, Q16: Pay equivalent to ability, Q15: Interest in the job, Q34: Attention of leadership, and Q33: Business training). The remaining 32 items had large factor loadings ranging from 0.508 to 0.845. These 32 items can be categorized into 7 common factors using PCA with Kaiser Normalization Varimax rotation. Cronbach’s alpha indicated that the items included in six common factors had good internal consistency, except for the job description factor. The first factor related to organizational management included 11 items. The second factor related to the internal environment included 4 items. The third factor related to the external environment included 5 items. The fourth factor related to working conditions included 4 items. The fifth factor related to competency behaviours included 3 items. The sixth factor relating to job rewards included 2 items. The seventh factor related to job description included 3 items.

**Table 3:** Questions included in each principal component and their factor loadings

Common factors	Cronbach’s alpha	Question No.	Variables	Loadings
Factor 1	0.951	Q44	Performance assessment	0.769
Organizational management (11 items)		Q40	Effective rules and regulations	0.761
		Q45	Incentive system	0.749
		Q42	Safety management system	0.748
		Q43	Task management system	0.748
		Q39	Income distribution system	0.746
		Q41	Human resources management	0.739
		Q38	Enterprise welfare	0.663
		Q37	Personal income	0.625
		Q31	Administration	0.611
		Q30	Logistical support	0.555
Factor 2	0.863	Q26	Relationship with colleagues	0.845
Internal environment (4 items)		Q27	Relationship with supervisors	0.828
		Q28	Cooperation within the department	0.750
		Q29	Cooperation with other departments	0.726
Factor 3	0.920	Q49	Role of government	0.797
External environment (5 items)		Q51	Resolution of medical disputes	0.779
		Q52	Health care system reform	0.766
		Q48	Media reports	0.730
		Q50	Supervision of care	0.704
Factor 4	0.781	Q24	Medical equipment	0.765
Working conditions (4 items)		Q23	Talented team	0.746
		Q25	Receipt of information	0.652
		Q22	Office conditions	0.508
Factor 5	0.696	Q19	Administration of medication	0.762
Competency behaviours (3 items)		Q21	Decision-making	0.710
		Q20	Group discussion	0.624
Factor 6	0.813			
Job rewards (2 items)		Q35	Job promotion	0.708
		Q36	Promotion in title	0.696
Factor 7	0.574	Q17	Challenging work	0.739
Job description (3 items)		Q18	Heavy workload	0.682
		Q47	Social recognition and respect	0.627

### Working conditions and job satisfaction

Statistical results for job satisfaction are shown in Table 4. In general, the mean score of job satisfaction was 3.41 (SD 0.68), which confirmed that certified physicians employed in PHCFs in rural Shandong Province were partially satisfied with their jobs. Respondents were most satisfied with the internal environment (median 4.50, QR 1.00) and their job description (median 4.33, QR 1.00). They were partially satisfied with competency behaviours (median 3.67, QR 1.33) and organizational management (mean 3.24, SD 0.95). However, respondents had a neutral attitude towards working conditions (median 3.00, QR 1.25) and job rewards (median 3.00, QR 2.00). In contrast, physicians were slightly dissatisfied with the external environment (median 2.80, QR 1.40).

**Table 4:** Descriptive statistics for job satisfaction in 7 dimensions

Dimension	Mean/Median	SD/Quartile range (QR)
Internal environment	4.50	1.00
Job description	4.33	1.00
Competency behaviours	3.67	1.33
Organizational management^a^	3.24	0.95
Working conditions	3.00	1.25
Job rewards	3.00	2.00
External environment	2.80	1.40
Overall job satisfaction^a^	3.41	0.68

^a^A Kolmogorov-Smirnov test indicated that the specific scale or item followed a normal distribution

### Demographic characteristics associated with job satisfaction among physicians

As shown in Table 5, the descriptive variables of age, educational background, technical title, monthly salary, form of employment, and years of service were found to be significantly associated with job satisfaction (*P* < 0.05), whereas the other four variables, including gender, marital status, hospital category, and hospital grade, were not significant. Additionally, Table 5 indicates that older physicians were significantly less satisfied with their jobs compared to younger ones (*P* < 0.01). Physicians over 46 years old had the lowest level of job satisfaction. Physicians with a higher level of education or senior technical title were significantly less satisfied with their jobs than those with a lower level of education or junior technical title (*P* < 0.01). Physicians with the title of chief physician or a master’s degree had the lowest satisfaction. Thus, retaining talented personnel is one of the greatest challenges for PHCFs and can easily lead to a serious “brain drain.” Moreover, three-quarters of physicians’ monthly salaries were between 2000 and 4000 CNY (*P* < 0.01). Only 4% of physicians earned more than 5000 CNY a month. Physicians who were temporary/casual employees were more satisfied with their jobs than those who were permanent full-time employees (*P* < 0.05). Moreover, physicians with numerous years of service were significantly less satisfied with their jobs (*P* < 0.05). There was no significant difference in job satisfaction for physicians at CHs and THs, and the same was true for the hospital grade.

**Table 5:** Demographic characteristics associated with job satisfaction among physicians

Variables	Groups	*N*	Score	F	*P*
Gender	Male	310	3.40	0.034	0.853
	Female	185	3.42		
Age	Younger than 25	16	3.69	5.237	0.001
	26–35	159	3.52		
	36–45	260	3.38		
	Over 46	60	3.16		
Marital status	Not married	40	3.49	0.569	0.451
	Married	455	3.40		
Educational background	Technical school graduate	25	3.49	3.276	0.021
	Junior college graduate	132	3.56		
	University graduate	306	3.35		
	Recipient of a master’s degree	32	3.29		
Technical title	Medical Assistant	47	3.68	3.674	0.006
	Resident Physician	132	3.49		
	Attending Physician	253	3.34		
	Associate Chief Physician	57	3.33		
	Chief Physician	6	3.07		
Monthly salary (CNY)	Less than 2000	36	3.81	3.647	0.006
	2001-3000	163	3.37		
	3001-4000	207	3.39		
	4001-5000	69	3.34		
	Over 5001	20	3.46		
Form of employment	Permanent full-time	429	3.38	5.094	0.024
	Temporary/casual	66	3.58		
Years of service	Fewer than 5	103	3.49	2.706	0.020
	6–10	88	3.58		
	11–15	77	3.38		
	16–20	138	3.36		
	21–25	59	3.27		
	More than 26	30	3.21		
Hospital category	CH	204	3.37	1.298	0.255
	TH	291	3.44		
Hospital grade	Grade I hospital	282	3.44	1.664	0.198
	Grade II hospital	213	3.36		

### Multivariate linear regression analysis of multi-item scales

The results of multivariate linear regression analysis of overall job satisfaction are shown in Table 6. The 7 multi-item scales were entered as regressors in a multivariate linear regression model estimated using ordinary least squares (OLS). During analysis, the 7 factors were found to be significantly associated with job satisfaction (*P* < 0.05). From the highest to the lowest regression coefficient, those factors were organizational management, the external environment, the internal environment, working conditions, job rewards, competency behaviours, and job description. Organizational management and the external environment were closely realted to job satisfaction. Organizational management was the most important factor for job satisfaction. This means that better hospital management can imporve physicians’ job satisfaction. The second important factor was the external environment. This finding is enlightening since job satisfaction among physicians could be improved through intensifying reforms in the medical system, strengthening supervision of care, improving the physician-patient relationship, promoting media reporting, resolving medical disputes, and other efforts to create a favourable external environment.

**Table 6:** Multivariate linear regression analysis of multi-item scales

Factors for job satisfaction	Unstandardized coefficients		Standardized coefficients	T	P	95.0% confidence interval for beta	
	Beta	Std. error	Beta			Lower bound	Upper bound
(Constant)	3.409	0.004		968.840	0.000	3.402	3.416
Factor 1 for organizational management	0.433	0.004	0.635	122.923	0.000	0.426	0.440
Factor 3 for the external environment	0.292	0.004	0.429	83.015	0.000	0.285	0.299
Factor 2 for the internal environment	0.242	0.004	0.355	68.762	0.000	0.235	0.249
Factor 4 for working conditions	0.229	0.004	0.335	64.885	0.000	0.222	0.235
Factor 6 for job rewards	0.200	0.004	0.293	56.676	0.000	0.193	0.207
Factor 5 for competency behaviours	0.172	0.004	0.252	48.767	0.000	0.165	0.179
Factor 7 for job description	0.074	0.004	0.109	21.069	0.000	0.067	0.081

## Discussion

The current results confirm that primary care physicians in Shandong Province are only slightly satisfied with their work. This is slightly higher than the job satisfaction of primary care employees as reported in the domestic and foreign literature [14, 23, 30]. The current results indicated that primary care physicians are slightly more satisfied with their jobs than moderately so, which is consistent with the resluts of a similar study in China [14]. Additionally, physicians with a higher level of education, senior technical title or numerous years of service were less satisfied than other physicians. In contrast, the primary health care physicians working in Lithuania for 30–39 years were most satisfied with their jobs [30].

Moreover, physicians who are senior specialists or younger tend to quit due to lower levels of job satisfaction, as was noted in a previous study [32]. PHCFs face the ongoing challenge of a shortage of personnel. A previous study indicated that good relations with co-workers and support from superiors led to a sense of satisfaction, and that social support was the most consistent and potent predictor of job satisfaction [35]. The current analysis identified 7 factors that were associated with the job satisfaction among physicians in PHCFs. Among them, the internal environment and job description factors scored the highest. This indicates that primary care physicians have a harmonious relationship with their colleagues and organizations, and that they are engaged in challenging and valuable work. In contrast to the two factors mentioned, the external environment was the factor with the lowest average score. This indicated that respondents were slightly dissatisfied with the current health care system reforms. As these reforms intesfy physicians flet a great deal of pressure from the external environment, in terms of medical disputes, media reports, and supervision of care, as well as reform of the medical system itself.

Primary care physicians were partially satisfied with competency behaviours and organizational management, but neutral on working conditions and job rewards. These findings suggest that physicians in PHCFs did not find their job rewarding in terms of career development or a promotion in technical title.

Last but not least, multivariate linear regression analysis indicated that organizational management and the external environment were the most important factors associated with job satisfaction. That said, the average score for both factors was almost in the middle of the scale. The external environmental had only a minimun score of 2.80. In other words, it is urgent and necessary to enhance organizational management and improve the external environment to increase job satisfaction among primary health care physicians. It is certainly high time that the Government considered giving PHCFs greater operational and staffing autonomy to stimulate vitality and create an effective development environment [24, 27].

Shandong proposed a model in which “CHs are leaders, THs are hubs, and VCs are the foundation” to enhance regional health consortia (RHCs). A previous study found that the GOC invested large amounts of money in PHCFs [5]. Although the Shandong provincial government has gradually increased its investment in PHCFs, it has not completely changed circumstances since both PHCFs and physicians are still heavily dependent on income from medical services. Therefore, medical reform in Shandong has not effectively increased physicians’ satisfaction.

Although the GOC has issued a series of policies, some have not yet been adequately implemented at the local level. The Government should comprehensively and extensively examine the effectiveness of its policies because the fate of primary health care physicians is bound up with these policies. In turn, good policies and physicians’ active participation will contribute to the success of health care reforms. The Government should protect the legitimate rights and interests of physicians when devising medical reforms in terms of supervision of care, media reporting, and the handling of disputes.

In addtion, the internal environment and working conditions are secondary contributors to job satisfaction. Motivating and retaining qualified employees is crucial to furthering the success of service organizations [21]. This indicates the importance of building talented teams and improving medical equipment. Only job description contributed the least to overall satisfaction.

In 2017, the Department of Human Resources and Health Commission of Shandong Province issued a notice on the granting of senior technical titles to health technicians at the local level in order to gradually establish a system of classification and evaluation. This notice stipulates that physicians with a local senior professional title could only be hired by PHCFs while those with a standardized provincial senior title could be employed in health care facilities in Shandong Province. The purpose of that change was to encourage exceptional personnel to continue working in PHCFs. This will greatly increase the remuneration of medical staff at PHCFs, enhance their job satisfaction, and also attract other talented personnel.

### Limitations of this study

This study had several limitations. First, during this cross-sectional study investigation, the respondents may be reluctant to give correct answers to some sensitive questions, which may result in report bias. At the same time, there may be bias in the random selection control of objects. Second, this study only examined the job satisfaction of physicians and there are no historical data for comparison. With the intensifying of China’s medical reform, it is of great value to enrich and verify the current results through in depth interviews with administrators and physicians in PHCFs, and further investigation can be carried out to establish time series data for comparison in the future. Further conclusions may be drawn if the study encompassed all medical staff, including administrators and technicians. Third, this scale has rather unsatisfactory internal consistency reliability. Lastly, the instrument developed in this study was not adequately validated and the structural validity of the questionnaire was not assessed. Its reliability and validity need to be assessed further in the future.

## Conclusion

The current study has investigated the current job satisfaction of primary health care physicians in Shandong Province and it has identified factors influencing that satisfaction. Since recent healthcare reforms, the job satisfaction of primary health care physicians has changed little compared to that of physicians in other provinces in China [31, 33, 45]. The current findings reflect the needs to recognize the influence of variables (age, educational background, technical title, monthly salary, form of employment, and years of service) on job satisfaction. Sufficient attention should be paid to the lower level of satisfaction of physicians with a higher level of education, senior technical title or numerous years of service based on their current realistic situation. Factors such as organizational management and the external environment have the greatest impact on the job satisfaction of health care workers. Numerous recommendations may be considered to enhance organizational management and improve the external environment. The Government should enhance the formulation, implementation, and evaluation of policies to ensure that beneficial policies can be implemented and have their intended effect. Giving PHCFs moderate autonomy may be an avenue for further efforts.

## Additional file

Additional file 1: Job satisfaction questionnaire. (DOC 97 kb)

## Abbreviations

ANOVA: Analysis of variance
CH: County hospital
CNY: Chinese yuan
EFA: Exploratory factor analysis
GOC: Government of China
OLS: Ordinary least squares
PCA: Principal component analysis
PHCF: Primary health care facility
QR: Quartile range
RHC: Regional health consortia
SD: Standard deviation
TH: Township hospital
THC: Township health centre
VC: Village clinic
