# Supplementary material for: Job satisfaction of certified primary care physicians in rural Shandong Province, China: a cross-sectional study
Source: BMC Health Serv Res. 2019 Jan 28;19:75. doi: 10.1186/s12913-019-3893-8 (PMC6350299; doi:10.1186/s12913-019-3893-8)
Supplement: Supplementary file 1 — Job satisfaction questionnaire. (DOC 97 kb) [file 12913_2019_3893_MOESM1_ESM.doc]

**Job Satisfaction Questionnaire**

Notice: We carry out this questionnaire survey to understand the working status of certified physicians in primary health care facilities, to ascertain the impact factors influencing job satisfaction, and to provide reference for decision making to improve physicians’ working environment. This questionnaire, consisting of 48 items in two parts, is used to evaluate the job satisfaction of certified primary care physicians. This survey is for statistical analysis only, and it is anonymous. None of your privacy will be involved, and we will keep all your information confidential.

If you have any objections, you can refuse to fill in the survey.

Thank you!

1. PartⅠ

Notice : please fill in the value or mark your personal choice in the options section.

| **Characteristics** | **Options** |
| --- | --- |
| Hospital category | 1. Township hospital ② County hospital |
| Hospital grade | 1. GradeⅠhospital ②GradeⅡhospital |
| Gender | 1. Male ② Female |
| Age | _______years old |
| Marital status | 1. Not married ② Married |
| Educational background | 1. Technical school graduate ②Junior college graduat ③ University graduate ④ Recipient of a master’s degree |
| Technical title | 1. Medical Assistant ② Resident Physician ③ Attending Physician ④ Associate Chief Physician ⑤ Chief Physician |
| Monthly salary (CNY) | 1. Less than 2,000 ② 2,001-3,000 ③ 3,001-4,000 ④ 4,001-5,000 ⑤ Over 5,001 |
| Form of employment | 1. Permanent full-time ② Temporary/casual |
| Years of service | _______years |

Part Ⅱ

Notice: those items are measured on a 5-point scale ranging from 1 to 5, where “1” represents extremely dissatisfied, “2” represents dissatisfied, “3” represents neither satisfied nor dissatisfied, “4” represents satisfied, and “5” represents extremely satisfied.

Please mark your personal choice in the options section.

| **Items** | **Options** | | | | |
| --- | --- | --- | --- | --- | --- |
| Interest in the job | 1 | 2 | 3 | 4 | 5 |
| Pay equivalent to ability | 1 | 2 | 3 | 4 | 5 |
| Challenging work | 1 | 2 | 3 | 4 | 5 |
| Heavy workload | 1 | 2 | 3 | 4 | 5 |
| Administration of medication | 1 | 2 | 3 | 4 | 5 |
| Group discussion | 1 | 2 | 3 | 4 | 5 |
| Decision-making | 1 | 2 | 3 | 4 | 5 |
| Office conditions | 1 | 2 | 3 | 4 | 5 |
| Talented team | 1 | 2 | 3 | 4 | 5 |
| Medical equipment | 1 | 2 | 3 | 4 | 5 |
| Receipt of information | 1 | 2 | 3 | 4 | 5 |
| Relationship with colleagues | 1 | 2 | 3 | 4 | 5 |
| Relationship with supervisors | 1 | 2 | 3 | 4 | 5 |
| Cooperation within the department | 1 | 2 | 3 | 4 | 5 |
| Cooperation with other departments | 1 | 2 | 3 | 4 | 5 |
| Logistical support | 1 | 2 | 3 | 4 | 5 |
| Administration | 1 | 2 | 3 | 4 | 5 |
| Competence improved | 1 | 2 | 3 | 4 | 5 |
| Business training | 1 | 2 | 3 | 4 | 5 |
| Attention of leadership | 1 | 2 | 3 | 4 | 5 |
| Job promotion | 1 | 2 | 3 | 4 | 5 |
| Promotion in title | 1 | 2 | 3 | 4 | 5 |
| Personal income | 1 | 2 | 3 | 4 | 5 |
| Enterprise welfare | 1 | 2 | 3 | 4 | 5 |
| Income distribution system | 1 | 2 | 3 | 4 | 5 |
| Effective rules and regulations | 1 | 2 | 3 | 4 | 5 |
| Human resources management | 1 | 2 | 3 | 4 | 5 |
| Safety management system | 1 | 2 | 3 | 4 | 5 |
| Task management system | 1 | 2 | 3 | 4 | 5 |
| Performance assessment | 1 | 2 | 3 | 4 | 5 |
| Incentive system | 1 | 2 | 3 | 4 | 5 |
| Recognition by the patient | 1 | 2 | 3 | 4 | 5 |
| Social recognition and respect | 1 | 2 | 3 | 4 | 5 |
| Media reports | 1 | 2 | 3 | 4 | 5 |
| Role of government | 1 | 2 | 3 | 4 | 5 |
| Supervision of care | 1 | 2 | 3 | 4 | 5 |
| Resolution of medical disputes | 1 | 2 | 3 | 4 | 5 |
| Health care system reform | 1 | 2 | 3 | 4 | 5 |

That’s the end of the questionnaire.

Thanks for your support and cooperation!
